# Tri-Layered Vascular Grafts Guide Vascular Cells’ Native-like Arrangement

**DOI:** 10.3390/polym14071370

**Published:** 2022-03-28

**Authors:** Xingyu Yuan, Wen Li, Bin Yao, Zhao Li, Deling Kong, Sha Huang, Meifeng Zhu

**Affiliations:** 1 School of Medicine, Nankai University, Tianjin 300071, China; yxy950210@163.com; 2 College of Life Science, State Key Laboratory of Medicinal Chemical Biology, Key Laboratory of Bioactive Materials of Ministry of Education, Nankai University, Tianjin 300071, China; liwen1182613400@163.com (W.L.); kongdeling@nankai.edu.cn (D.K.); 3 Research Center for Tissue Repair and Regeneration, The Medical Innovation Research Department, PLA General Hospital and PLA Medical College, Beijing 100853, China; hongyaobin1212@163.com (B.Y.); lizhao0215@163.com (Z.L.)

**Keywords:** biomimetic, multilayer structure, smooth surface, aligned microfiber, random microfiber

## Abstract

Bionic grafts hold great promise for directing tissue regeneration. In vascular tissue engineering, although a large number of synthetic grafts have been constructed, these substitutes only partially recapitulated the tri-layered structure of native arteries. Synthetic polymers such as poly(l-lactide-*co*-ε-caprolactone) (PLCL) possess good biocompatibility, controllable degradation, remarkable processability, and sufficient mechanical strength. These properties of PLCL show great promise for fabricating synthetic vascular substitutes. Here, tri-layered PLCL vascular grafts (TVGs) composed of a smooth inner layer, circumferentially aligned fibrous middle layer, and randomly distributed fibrous outer layer were prepared by sequentially using ink printing, wet spinning, and electrospinning techniques. TVGs possessed kink resistance and sufficient mechanical properties (tensile strength, elastic modulus, suture retention strength, and burst pressure) equivalent to the gold standard conduits of clinical application, i.e., human saphenous veins and human internal mammary arteries. The stratified structure of TVGs exhibited a visible guiding effect on specific vascular cells including enhancing endothelial cell (EC) monolayer formation, favoring vascular smooth muscle cells’ (VSMCs) arrangement and elongation, and facilitating fibroblasts’ proliferation and junction establishment. Our research provides a new avenue for designing synthetic vascular grafts with polymers.

## 1. Introduction

Constructing grafts mimicking the defining features of native tissues is a promising strategy for regenerative medicine [1,2,3,4]. Native arteries consist of a tri-layered structure from inner to outer with distinct patterns: (i) an intima composed of a smooth longitudinally oriented endothelial cell (EC) monolayer; (ii) a media composed of multiple concentric layers of circumferentially aligned vascular smooth muscle cells (VSMCs); (iii) an adventitia mainly composed of randomly distributed fibroblasts [5]. A heterogeneous structure is the prerequisite of mechanical integrity, vasoconstriction, vasodilation, anti-coagulant properties, etc. [6]. Therefore, preparing vascular grafts with a tri-layered structure mimicking native arteries will contribute to vascular tissue engineering and regeneration.

Currently, a large number of techniques including electrospinning, phase separation, 3D printing, salt leaching, photoetching, braiding, etc., have been utilized to construct synthetic vascular grafts [7,8,9,10]. However, these grafts only partially recapitulated the architecture of arteries. Our previous studies proved that circumferentially aligned fibers promoted VSMCs’ oriented regeneration and the smooth membrane enhanced the ECs’ monolayer formation [11,12]. However, these two grafts lacked a tri-layered structure capable of simultaneously guiding the native-like arrangement of three principal vascular cells (ECs, VSMCs, and fibroblasts) [13,14].

In addition to structural design, the mechanical properties of biomaterials are also an important element to be considered in the preparation of vascular grafts. These properties not only determine the processibility, but also affect the hemocompatibility and even the final regeneration effect of scaffolds. For example, the surface mechanical properties of substrates enable mediating platelet adhesion, spreading, and activation, and the greater stiffness of the materials, the greater the platelets’ adhesion and activation are [15,16,17]. The mechanical properties of the scaffolds also have essential effects on macrophage polarization, contractile VSMCs’ determination, and ECM deposition, which may influence the vascular remodeling results [18,19]. In general, vascular scaffolds should be easy to handle, sutured, and possess adequate kink resistance and compliance [20]. Poly(l-lactide-*co*-ε-caprolactone) (PLCL) is a copolymer of poly(l-lactic acid) and poly(ε-caprolactone). The mechanical properties of this material depend on the ratio of lactic acid and caprolactone and can therefore be tailored for different applications: from rigid to elastic. In recent years, PLCL has been widely used to prepare vascular scaffolds due to its elasticity, biocompatibility, and degradability [21,22,23]. The study of Zhang et al. showed that the compliance of well-designed three-layered vascular grafts containing PLCL was similar to that of human blood vessels [21].

In view of this, tri-layered vascular grafts (TVGs) containing layer-specific guiding structures for unique vascular cells were prepared with PLCL copolymer, a promising scaffold material with excellent processability and sufficient mechanical strength [11]. The inner layer with a smooth surface was generated by ink printing, favoring endothelialization. The middle layer composed of circumferentially aligned microfibers was fabricated by wet spinning, benefiting VSMCs’ oriented regeneration. The outer layer consisting of randomly distributed fibers was prepared by electrospinning, contributing to fibroblasts’ uniform distribution. Furthermore, the mechanical properties of the grafts were evaluated, and the guiding effects of the specific topological structure in each layer of the grafts on ECs, VSMCs, and fibroblasts were characterized.

## 2. Materials and Methods

### 2.1. Graft Fabrication

TVGs were fabricated by following techniques described before [11,12]. First, the inner layer was prepared by ink printing. PLCL (50:50; Jinan Daigang, Jinan, China) dissolved (5%, *w*/*v*) in hexafluoroisopropanol (HFIP; Aladdin, Shanghai, China) was extruded at 1.5 mL/h and wrapped on a 2 mm-diameter rotating rod. Second, the middle layer was fabricated by wet spinning. PLCL solution (10%, *w*/*v* in HFIP) was squeezed into a coagulation bath of 95% ethanol at 2 mL/h and collected on the rotating rod with an inner layer for 13 min after turning into solid filaments. Third, the outer layer was prepared by electrospinning with the following conditions: 10% PLCL solution, 2 mL/h, 21 G nozzle, 16 kV, nozzle collector distance of 10 cm, and electrospinning on the middle layer for 3.5 min. Finally, residual solvent was removed under a vacuum. The control group including wet spinning grafts (WSGs) and electrospinning grafts (ESGs) were prepared in 16 min and 30 min, respectively, the conditions of which were consistent with the middle and outer layer of TVGs. Wet spinning and electrospinning membranes were developed with a 2 cm- and 10 cm-diameter rotating mandrel for 40 min and 2 h. Smooth surface substrates were prepared by casting 10% PLCL solution in a flat glass dish.

### 2.2. Structure Characterization

A scanning electron microscope (SEM; Quanta 200; FEI, Hillsboro, OR, USA) was used to observe structural features of the grafts at 15 kV. Fiber diameter and layer thickness were manually measured using Image-Pro Plus 6.0 (IPP; Media Cybernetics, Rockville, MD, USA) based on the SEM images.

### 2.3. Roughness Characterization

Roughness measurements were performed using a white light interferometer (NANO SYSTEM, NV-2700, Daejeon, Korea). The topological data of the smooth inner layer were obtained from the measurements of three different membranes.

### 2.4. Fiber Density Measurements

Six SEM images were selected from three independently fabricated samples for each group. A reference line perpendicular to the majority of the fibers was drawn on the SEM image, and all fibers crossing the reference line were counted with IPP. The density is presented as the number of fibers per millimeter for each group [24].

### 2.5. Mechanical Tests

Tensile tests were carried out on a uniaxial tensile testing machine (Instron 3345, Boston, MA, USA) (*n* = 6) [11]. For the longitudinal direction, samples of 3.0 cm in length were stretched at a speed of 0.16 mm/s until rupture. For the radial direction, two homemade steel rings were passed through the lumen of samples (0.3 cm in length) and pulled radially at the same rate until rupture. Young’s modulus was calculated based on the slope of the stress–strain curve. Suture retention strength was collected from a film of 2.0 cm (L) × 0.5 cm (W) generated from the tubular samples [11]. Sutures (6-0; Jinhuan, China) were stretched at 0.16 mm/s until the sample rupture, and the maximum load was recorded. Burst pressure was obtained by filling the scaffolds (1.0 cm in length) with Vaseline [11]. Then, a continuous filling rate of nitrogen was employed at 0.1 mL/min, and the burst pressure was recorded until a leakage occurred. Porosity was calculated with Archimedes’ principle using a pycnometer (*n* = 6) [11].

### 2.6. Cell Culture and Evaluation

Mouse embryo fibroblast cell line NIH/3T3 and human umbilical vein endothelial cells (HUVECs) were obtained from ScienCell (Carlsbad, CA, USA). Rat vascular smooth muscle cell line A10 was obtained from the American Type Culture Collection (ATCC). Dulbecco’s Modified Eagle’s Medium-High Glucose (DMEM-H) and fetal bovine serum (FBS) were received from Gibco (Carlsbad, CA, USA). Endothelial Cell Medium was acquired from ScienCell.

To evaluate the guiding effects of TVGs, HUVECs (2 × 10^4^ cells/cm^2^), A10 cells (1 × 10^4^ cells/cm^2^), and NIH/3T3 cells (6 × 10^3^ cells/cm^2^) were seeded on the smooth surface and wet spun and electrospun membranous scaffolds, respectively, for 1 d, 3 d, and 5 d. Cells were identified by staining of Phalloidin-iFluor 488 (Abcam, Cambridge, UK; ab176753) and 4’,6-diamidino-2-phenylindole (DAPI; Invitrogen, Waltham, MA, USA). Fluorescent images were captured by a laser scanning confocal microscope (Leica, Wetzlar, Germany). The spreading area and density of cells were analyzed by IPP software.

### 2.7. Cellular Alignment and Shape Evaluation

The morphology response of the vascular cells to the contact guidance of each layer in TVGs was quantitatively evaluated in terms of the angle orientation and the shape index (SI). As shown in Figure 4d, the cell angle orientation was defined as the angle between the major axis of cells and the fiber direction. The angle of 0° represents a cell perfectly aligned with the fiber, and ±90° represents a cell perpendicular to the fiber. Fifty individual cells were analyzed to statistically evaluate the cellular orientation angles [25]. At least three independent replicates were performed for each type of vascular cell.

The morphology of the vascular cells was evaluated by the SI using the following equation:

SI = 4πA/P^2^ (1)

where A represents the cell area and P represents the cell perimeter. The value of the SI ranges from 0 (a straight line) to 1 (a perfect circle), which reveals the degree of elongation of a cell. Sixty individual cells were analyzed to statistically evaluate the cell morphology [25].

### 2.8. Statistical Analysis

All quantitative results were obtained from at least three samples and from three in-dependent experiments. All data were analyzed with GraphPad Prism 7.0 and presented as the mean ± standard deviation (s.d.). The comparison between the two groups was performed by the unpaired two-tailed *t*-test. For multiple-group comparison, one-way ANOVA with Tukey’s multiple comparison test was used. The group significance level is denoted as * *p* < 0.05, ** *p* < 0.01, *** *p* < 0.001, and **** *p* < 0.0001.

## 3. Results and Discussion

### 3.1. Morphological Observation

**Figure 1:**
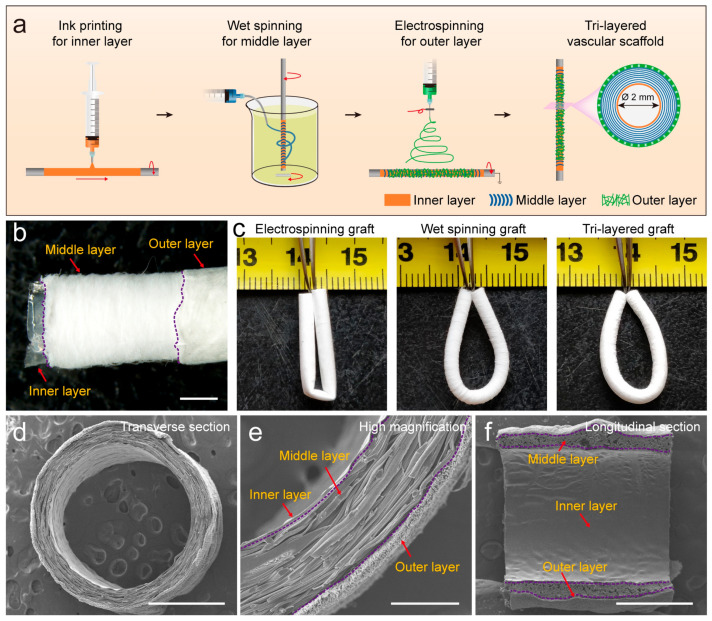
Fabrication and structure of TVGs. (**a**) Schematic illustration showing the preparation process. (**b**) Gross morphology and (**c**) kink resistance. (**d**–**f**) SEM images representing the macro-/micro-structure in the cross and longitudinal section. Scale bars: (**b**,**d**), 1 mm; (**e**) 200 μm; (**f**) 1 mm.

**Figure 2:**
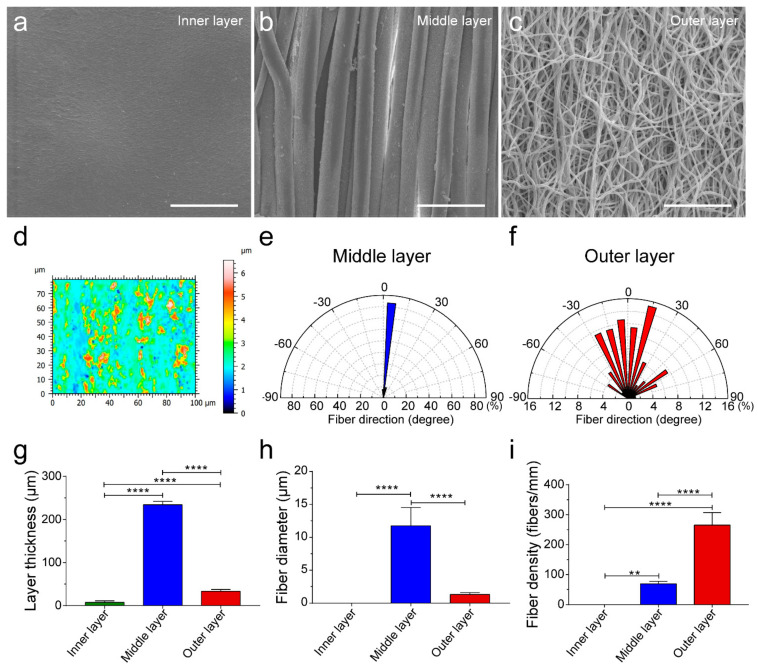
Characterization of the smooth inner layer, circumferentially aligned fibrous middle layer, and randomly distributed fibrous outer layer of TVGs. (**a**–**c**) SEM images showing the topological structure of the inner layer (**a**), middle layer (**b**), and outer layer (**c**) of TVGs. (**d**) White light interferometer images representing the low roughness of the smooth inner layer. The color scale indicates the relative height of the smooth film surface. The smaller the color difference shown in the image, the smoother the film surface is. (**e**,**f**) The wind rose diagrams indicate the highly organized and aligned fibers within the circumferentially aligned fibrous middle layer, whereas the arbitrary orientation of individual fibers was observed in the randomly distributed fibrous outer layer. (**g**–**i**) Quantitative analysis of the layer thickness (*n* = 3), fiber diameter (*n* = 100), and fiber density (*n* = 6) of TVGs using SEM images. The results are expressed as the mean ± s.d., ** *p* < 0.01 and **** *p* < 0.0001. Scale bars: (**a**–**c**) 50 μm.

By mimicking the intima, media, and adventitia of arteries, TVGs were fabricated by sequentially using ink printing, wet spinning, and electrospinning techniques (Figure 1a,b). Control groups merely containing circumferentially aligned filaments (WSGs) or randomly distributed fibers (ESGs) were prepared with the wall thickness close to TVGs (Figure 1c). Kink resistance is one of the critical flexible features of vascular grafts to fit various grafting conditions, in particular in the anatomic location with continuous bending and moving, i.e., carotid artery bypass, axillary femoral, and popliteal regions’ transplantation [26,27]. In these areas, the joint motion may cause the collapse of implants [18]. Circumferentially aligned fibers in TVGs and WSGs guarantee the scaffolds’ unkinking after being folded at 180°, while ESGs form dead folds easily (Figure 1c). SEM images showed that TVGs had an apparent and tightly bonded tri-layered structure (Figure 1d–f). From inner to outer, the stratified structure consisted of a smooth surface film (Figure 2a,d), circumferentially aligned fibers (fiber diameter = 12.0 ± 2.7 μm; Figure 2b,e), and randomly distributed fibers (fiber diameter = 1.4 ± 0.3 μm; Figure 2c,f) and had a layer thickness of 7.6 ± 3.3 μm, 234.4 ± 7.5 μm, and 33.0 ± 4.6 μm, respectively (Figure 2g). Furthermore, TVGs presented a lumen diameter of 1.9 ± 0.1 mm, which was slightly smaller than that of the rotating rod (2.0 mm).

### 3.2. Mechanical Analysis

**Figure 3:**
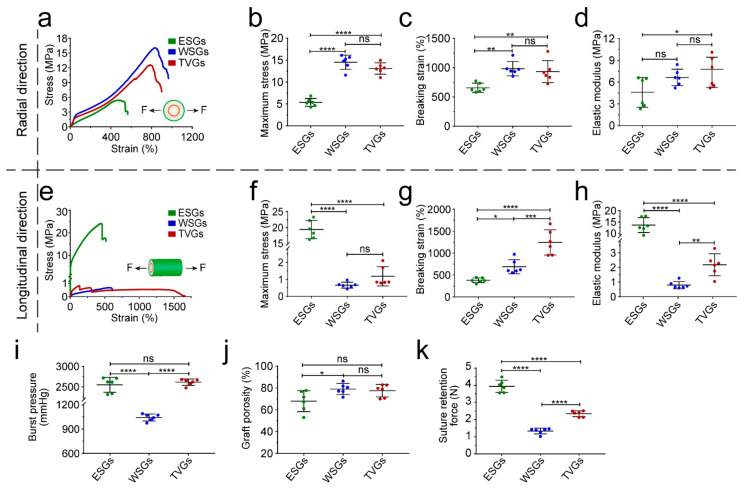
Mechanical analysis of various grafts. (**a**–**h**) Stress–strain curves (**a**,**e**), maximum stress (**b**,**f**), breaking strain (**c**,**g**), and elastic modulus (**d**,**h**) in the radial and longitudinal direction (*n* = 6). Arrows indicate the tensile direction. (**i**–**k**) Burst pressure (**i**), porosity (**j**), and suture retention force (**k**) of grafts (*n* = 6). The results are expressed as the mean ± s.d., * *p* < 0.05, ** *p* < 0.01, *** *p* < 0.001, and **** *p* < 0.0001. ns, not significant.

Artificial vascular grafts with enough mechanical strength can resist blood pressure continuously [28,29,30,31]. In the radial direction, the maximum stress, breaking strain, and modulus of TVGs were comparable to WSGs, while largely higher than those of ESGs, mainly due to the existence of circumferentially aligned fibers (Figure 3a–d). In the longitudinal direction, those tensile parameters of TVGs showed a minor difference from WSGs, while exhibiting a huge difference from ESGs (Figure 3e–h). Besides, the burst pressure of TVGs was much higher than that of the control groups and human saphenous veins (1680 ± 307 mmHg), considered the gold standard for vascular bypasses, probably attributed to the synergistic sealing effect of the three-layered structure (Figure 2h,i and Figure 3i,j). Furthermore, the suture retention strength of TVGs was significantly higher than that of WSGs and human internal mammary arteries (0.56 ± 0.19 N), another gold standard conduit, indicating that the mechanical strength of TVGs meets the clinical application requirements (Figure 3k) [32].

### 3.3. Cell Behavior Detection

**Figure 4:**
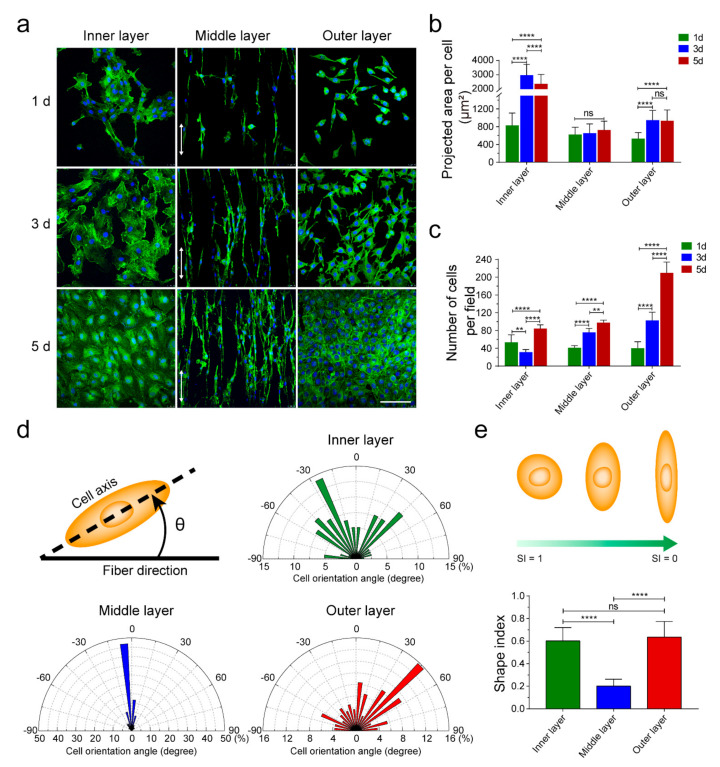
Guiding effects of the topological structure in each layer on vascular cells. (**a**) Phalloidin-iFluor 488 and DAPI staining showing ECs’, VSMCs’, and fibroblasts’ morphology on the inner, middle, and outer layer. White arrows indicate the fiber direction. (**b**,**c**) Spreading area and density of vascular cells cultured on different layers at Days 1, 3, and 5. (**d**) Orientation angle analysis of the vascular cells cultured on smooth surface substrates (inner layer) and aligned (middle layer) and randomly (outer layer) distributed fibrous scaffolds, respectively (*n* = 80). Schematic showing the orientation angle of cells. (**e**) The statistical analysis of the SI of the vascular cells cultured on smooth surface substrates (inner layer) and aligned (middle layer) and randomly (outer layer) distributed fibrous scaffolds, respectively (*n* = 60). Schematic showing changes in cell morphology with decreasing SI. The results are expressed as the mean ± s.d., ** *p* < 0.01 and **** *p* < 0.0001. ns, not significant. Scale bars: (**a**) 100 μm.

To detect the contact guidance of each layer in TVGs, vascular cells (HUVECs, A10 cells, and NIH/3T3 cells) were seeded onto smooth surface substrates and aligned and randomly distributed fibrous scaffolds, respectively, which is consistent with the topological structure of each layer of TVGs (Figure 4a). On the smooth surface substrates, HUVECs displayed a larger spreading area and higher cell density compared with the scattered irregular cell morphology at 1 d and 3 d (Figure 4b,c, inner layer). Furthermore, the cells rapidly organized into a fully confluent monolayer with a representative cobblestone appearance at 5 d (Figure 4a, inner layer), indicating an appropriate topology for rapid endothelialization [33,34,35]. On the aligned fibrous scaffolds, the density of A10 cells gradually increased from 1 d to 5 d (Figure 4c, middle layer). The cells grew longitudinally along the fiber axis (Figure 4a,d, middle layer) and displayed a slender spindle shape (SI = 0.2 ± 0.1) similar to the contractile morphology of VMSCs in arteries (Figure 4e, middle layer), indicating obvious guiding effects of the middle layer [36,37]. On the randomly distributed fibrous scaffolds, the spreading area of NIH/3T3 cells obviously increased at 3 d, and no visible change was noted at 5 d (Figure 4b, outer layer). The cell density steadily increased from 1 d to 5 d (Figure 4c, outer layer). Additionally, the cells extended numerous pseudopodia from the edge of the triangular or rectangular cell body at 3 d and assembled into a uniform cell sheet with established tight junctions at 5 d (Figure 4a, outer layer), indicating the suitable microenvironment for fibroblasts’ growth [38].

## 4. Conclusions

**Figure 5:**
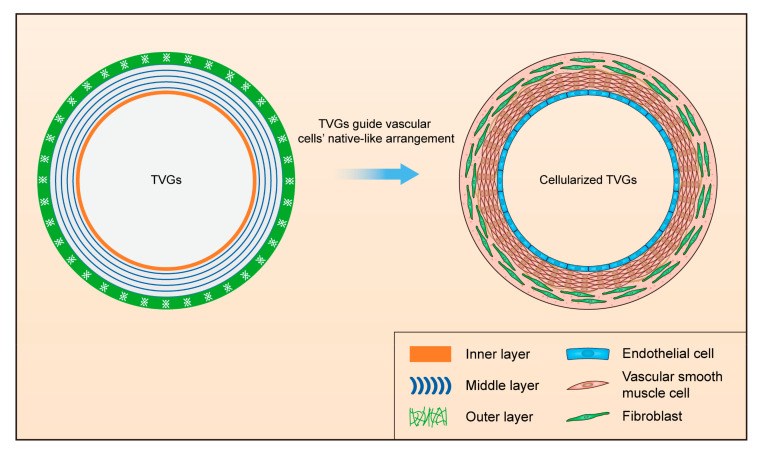
Schematic illustration of designing TVGs with a stratified biomimetic structure to guide the native-like arrangement of vascular cells.

An innovative tri-layered small-diameter vascular graft containing a smooth surface film, circumferentially aligned fibers, and randomly distributed fibers was prepared by combining ink printing, wet spinning, and electrospinning techniques (Figure 1a). TVGs possessed kink resistance and enough tensile strength for transplant applications compared with single-layered scaffolds. Vascular cells’ culture showed that the layer-by-layer bionic topology design in TVGs had a visible guiding effect on the cellular behaviors. The inner smooth surface film enhanced HUVECs’ spreading and monolayer formation. The middle circumferentially aligned fibers fully guided A10 cells’ orientation parallel with the fiber axis. The outer randomly distributed fibers improved NIH/3T3 cells’ proliferation and junction establishment. In summary, our strategy may be a promising alternative for designing engineered vascular scaffolds as TVGs provide satisfactory kink resistance, enough tensile strength, and a visible guiding effect on vascular cell behaviors (Figure 5).

## Data Availability

The data presented in this study are available upon request from the corresponding author.
